# Association between frequency of makeup use and depressive symptoms among community-dwelling older Japanese women

**DOI:** 10.1016/j.jarlif.2026.100073

**Published:** 2026-05-15

**Authors:** Yuto Kiuchi, Sho Nakakubo, Shinnosuke Nosaka, Yuka Misu, Kazuhei Nishimoto, Takamasa Gomi, Shu Nishikori, Kanon Abe, Tai Takemoto, Hiroyuki Shimada

**Affiliations:** a Frontier Research Center, POLA Chemical Industries, Inc., Yokohama, Kanagawa, 244-0812, Japan; b Department of Preventive Gerontology, Center for Gerontology and Social Science, Research Institute, National Center for Geriatrics and Gerontology, Obu, Aichi, 474-8511, Japan; c Department of Occupational Therapy, Faculty of Rehabilitation, Kobe Gakuin University, Kobe, Hyogo, 651-2180, Japan; d Department of Physical Therapy, Faculty of Health Sciences, Kyoto Tachibana University, Kyoto, Kyoto, 607-8175, Japan; e Cognitive Function Research, Aging Research (Partnership field), Nagoya University Graduate School of Medicine, Nagoya, Aichi, 466-8550, Japan; f Medical Science Division, Department of Medical Sciences, Graduate School of Medicine, Science and Technology, Shinshu University, Matsumoto, Nagano, 390-8621, Japan

**Keywords:** Older adults, Cosmetic behavior, Psychological functions, Aging

## Abstract

**Background:**

In the present study we aimed to examine the association between makeup use and depressive symptoms among community-dwelling older Japanese women.

**Methods:**

This cross-sectional analysis included 1183 community-dwelling older adults (mean age 76.3 ± 4.0 years). Depressive symptoms were assessed using the 15-item Geriatric Depression Scale, with scores ≥5 indicating depressive symptoms. Makeup use frequency was assessed by asking participants, “Do you usually wear makeup?” Response options were “Never,” “1–2 days per week,” “3–4 days per week,” and “≥5 days per week.”

**Results:**

The overall prevalence of depressive symptoms was 13.9% (*n* = 164). The prevalence of depressive symptoms by makeup use frequency was 19.5%, 14.7%, 13.9% and 10.2% in the never, 1–2 days/week, 3–4 days/week, and ≥5 days/week groups, respectively (p for trend <0.01). In adjusted logistic regression models, participants who used makeup ≥5 days/week had lower odds of depressive symptoms than those who never used makeup (odds ratio [OR] = 0.51, 95% confidence interval [CI] 0.34–0.77). Stratified analyses by social activity showed similar associations in the high (OR = 0.49, 95% CI 0.25–0.95) and the low (OR = 0.54, 95% CI 0.32–0.90) social activity group.

**Conclusion:**

Higher frequency of makeup use was associated with a lower prevalence of depressive symptoms among community-dwelling older Japanese women. This study used a cross-sectional design, which made it difficult to determine a causal relationship between makeup use and depressive symptoms. Future research should use longitudinal data to elucidate whether makeup use predicts the onset of depressive symptoms.

## 1 Introduction

Depressive symptoms are a common geriatric syndrome among older adult populations [1]. A meta-analysis reported a global prevalence of 35.1% for depressive symptoms among older adults [2]. These symptoms are associated with adverse health outcomes, including dementia [3], disability [4], and mortality [5]. Therefore, identifying protective factors for depressive symptoms is essential to maintaining quality of life in older adults.

Cosmetics are defined as articles applied to the human body for cleansing, beautifying, promoting attractiveness, or altering physical appearance [6], and makeup use is considered one component of broader appearance related self-care (e.g., personal grooming). Makeup use has been reported not only to enhance physical appearance but also to exert beneficial effects on psychological function. A cross-sectional study found that Brazilian women aged >30 years who used makeup had a lower prevalence of mild depression [7]. In addition, a randomized controlled trial demonstrated that encouraging more frequent makeup use resulted in sustained reductions in depressive symptoms among Brazilian women [8]. Interventional studies further suggest that beauty-related programs, including makeup use, skincare, and relaxation therapy, may improve cognitive function and social activity among older adults [9,10]. Collectively, these findings indicate that makeup use plays a role in cleansing, beautifying, promoting attractiveness, or altering physical appearance and may contribute to better psychological health, cognitive function, and social activity. Makeup use may not be a standalone treatment but may reflect broader behavioral and psychosocial patterns. However, cosmetic behavior differs across cultures and generations, and the association between makeup use frequency and depressive symptoms has not been examined among community-dwelling older Japanese women.

Social participation is also considered an important factor in preventing depression [11]. Prior studies have shown that social participation and interaction are associated with a lower risk of depressive symptoms among older adults [12,13]. Makeup use may also reflect social engagement and interpersonal interactions. Therefore, social activities should be considered when examining the association between makeup use and depressive symptoms.

We hypothesized that higher frequency of makeup use would be associated with a lower prevalence of depressive symptoms among community-dwelling older Japanese women and that this association would differ according to social activity level. Accordingly, this study investigated the association between makeup use and depressive symptoms in this population and conducted stratified analyses by social participation.

## 2 Methods

### 2.1 Participants

Potential participants were 2722 adults aged ≥70 years enrolled in a cohort study within the National Center for Geriatrics and Gerontology–Study of Geriatric Syndromes (NCGG-SGS) [14]. Exclusion criteria included male sex (*n* = 1356), disability in basic activities of daily living (*n* = 2), severe neurological disease (dementia, *n* = 1; Parkinson’s disease, n = 6), general cognitive impairment (Mini-Mental State Examination [MMSE] score <19; *n* = 6) [15], and missing data (*n* = 168). A total of 1183 participants were included in the analysis. All participants provided written informed consent, and the study protocol was approved by the Ethics Committee of the NCGG.

### 2.2 Measurements

#### 2.2.1 Frequency of makeup use

Makeup use frequency was assessed using the question “Do you usually wear makeup?” Response options were “Never,” “1–2 days per week,” “3–4 days per week,” and “≥5 days per week.”

#### 2.2.2 Depressive symptoms

Depressive symptoms were assessed using the 15-item Geriatric Depression Scale (GDS), which includes 15 “yes” or “no” items and yields scores from 0 to 15, with higher scores indicating greater symptom severity [16]. Participants with GDS scores ≥5 were classified as having depressive symptoms [17]. A score of 5 or higher was used to identify clinical depressive symptoms [18,19].

#### 2.2.3 Social activity

Social activity was assessed using the social score of the Active Mobility Index (AMI), which evaluates participants’ life space in relation to physical and social activities; the detailed protocol has been described previously [20]. Briefly, the AMI assesses three life-space levels during the past month: <1 km, 1–10 km, and ≥10 km from the participant’s residence. For each level, participants reported visit frequency per week (<once, 1–3, 4–6 days, or every day), purpose (primarily physical activity [such as walking or exercise], primarily daily chores or appointments [such as shopping or meeting people], or both equally), transportation mode (walking, bicycle, bus/train, car, or other), and extent of interaction with others (0, 1–2, 3–4, or ≥5 people). Life-space scores were calculated by multiplying the life-space level by visit frequency, and the AMI social score was derived by further incorporating social components, including purpose, transportation, and interaction with others. The social activity score ranged from 0 to 144, and participants with values below the median were classified as having low social activity.

#### 2.2.4 Covariates

In face-to-face interviews, participants provided information on sociodemographic characteristics (i.e., age, educational level and living alone), as well as clinical history (e.g., hypertension, diabetes, hyperlipidemia). Weight (kg) and height (m) were measured, and body mass index (BMI) was calculated as weight divided by height squared (kg/m^2^). Cognitive function was assessed using the MMSE [15]. Walking speed was evaluated on a 6.4-meter flat, straight walkway at a comfortable pace. After walking 2.0 m, participants crossed a marker indicating the start of the timed section, followed by a second marker 2.4 m later indicating the end of the timed section; the final 2.0 m allowed participants to decelerate and stop. Walking time was recorded in seconds using a stopwatch, and walking speed was calculated in meters-per-second (m/s) [21]. Work status was determined based on responses to the question, “Are you engaged in paid employment?” Participants who answered “yes” were classified as engaged in paid work, whereas those who answered “no” were classified as not engaged in paid work [22].

### 2.3 Statistical analysis

Statistical analyses were conducted using R version 4.2.1, with statistical significance set at *p* < 0.05. Descriptive statistics were used to summarize participant characteristics, with mean ± standard deviation reported for continuous variables and frequency and percent for categorical variables. Differences in participant characteristics and prevalence of depressive symptoms across makeup use frequency categories (Never, 1–2 days per week, 3–4 days per week, and ≥5 days per week) were examined using the Cochran–Armitage trend test for categorical variables and one-way analysis of variance for continuous variables. The Cochran–Armitage test for trends evaluated a linear trend by treating ordered exposure categories as continuous variables. The association between makeup use frequency and depressive symptoms was evaluated using multivariable logistic regression models, with the “never”-use group serving as the reference category. Results were expressed as odds ratios (ORs) with 95% confidence intervals (CIs). The present study selected the “never” group as the reference because it represents a clearly unexposed group, allowing clearer interpretation of the association between a higher frequency of makeup use and depressive symptoms. This study considered alternative groupings; however, because each frequency category included enough participants, we retained the original categories to avoid loss of information. Therefore, we did not conduct additional sensitivity analyses by using alternative groupings. To address both the adjustment factor and effect modifier, stratified analyses were performed based on the level of social activity. Model 1 was adjusted for age, medical history (hypertension, diabetes mellitus, and hyperlipidemia), education, paid work status, BMI, MMSE score, living alone, and gait speed. Model 2 included additional adjustment for social activity.

## 3 Results

The study sample included 1183 participants (mean age 76.3 ± 4.0 years). Makeup use frequency was distributed as follows: never, 313 participants (26.5%); 1–2 days/week, 177 participants (15.0%); 3–4 days/week, 166 participants (14.0%); and ≥5 days/week, 527 participants (44.5%). Participants with higher makeup use frequency demonstrated higher MMSE scores, faster gait speed, a greater proportion engaged in paid work, and higher levels of social activity compared with those with lower makeup use frequency (Table 1).

**Table 1:** Participant characteristics by frequency of makeup use.

Characteristic	Overall n = 1183	Never n = 313 (26.5%)	1–2 days/week n = 177 (15.0%)	3–4 days/week n = 166 (14.0%)	≥5 days/week n = 527 (44.5%)	p-value
Age, years	76.29 ± 4.04	76.55 ± 4.36	76.49 ± 4.28	75.99 ± 3.78	76.16 ± 3.83	0.385
Education, years	12.54 ± 2.20	12.34 ± 2.16	12.55 ± 2.40	12.77 ± 2.31	12.60 ± 2.11	0.194
Body Mass Index, kg/m^2^	22.36 ± 3.24	22.28 ± 3.27	22.55 ± 3.46	22.68 ± 3.22	22.24 ± 3.15	0.371
Hypertension						0.195
Absence	650 (54.9%)	183 (58.5%)	86 (48.6%)	94 (56.6%)	287 (54.5%)	
Presence	533 (45.1%)	130 (41.5%)	91 (51.4%)	72 (43.4%)	240 (45.5%)	
Hyperlipidemia						0.215
Absence	681 (57.6%)	194 (62.0%)	94 (53.1%)	91 (54.8%)	302 (57.3%)	
Presence	502 (42.4%)	119 (38.0%)	83 (46.9%)	75 (45.2%)	225 (42.7%)	
Diabetes mellitus						0.923
Absence	1069 (90.4%)	282 (90.1%)	161 (91.0%)	152 (91.6%)	474 (89.9%)	
Presence	114 (9.6%)	31 (9.9%)	16 (9.0%)	14 (8.4%)	53 (10.1%)	
Living arrangement						0.452
Living together	910 (76.9%)	239 (76.4%)	140 (79.1%)	134 (80.7%)	397 (75.3%)	
Living alone	273 (23.1%)	74 (23.6%)	37 (20.9%)	32 (19.3%)	130 (24.7%)	
Social activity						< 0.001
High active	575 (48.6%)	133 (42.5%)	77 (43.5%)	72 (43.4%)	293 (55.6%)	
Low active	608 (51.4%)	180 (57.5%)	100 (56.5%)	94 (56.6%)	234 (44.4%)	
Gait speed, m/s	1.19 ± 0.21	1.15 ± 0.23	1.19 ± 0.20	1.18 ± 0.21	1.21 ± 0.20	0.003
Paid work						< 0.001
Not working	929 (78.5%)	242 (77.3%)	160 (90.4%)	136 (81.9%)	391 (74.2%)	
Working	254 (21.5%)	71 (22.7%)	17 (9.6%)	30 (18.1%)	136 (25.8%)	
MMSE, score	27.11 ± 2.35	26.83 ± 2.55	27.39 ± 2.30	27.54 ± 2.18	27.05 ± 2.27	0.005

Continuous variables are presented as means ± SD, and categorical variables are presented as numbers (%). Continuous variables were analyzed using one-way analysis of variance, and categorical variables were analyzed using the Cochran–Armitage test. SD = standard deviation, MMSE = Mini-Mental State Examination.

**Fig. 1:**
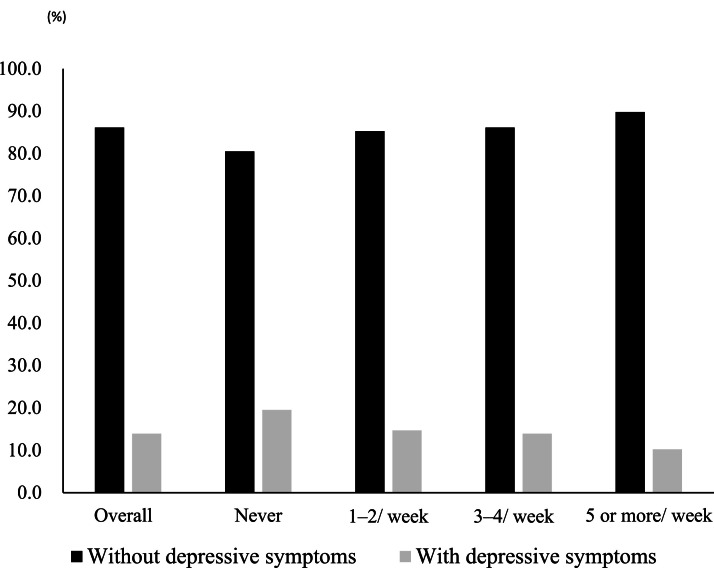
Prevalence of depressive symptoms by frequency of makeup use.

Fig. 1 illustrates the association between makeup use frequency and the prevalence of depressive symptoms. The overall prevalence of depressive symptoms was 13.9% (*n* = 164). Prevalence by makeup use frequency was never, 19.5% (*n* = 61), 14.7% (*n* = 26), 13.9% (*n* = 23), and 10.2% (*n* = 54), in the never, 1–2 days/week, 3–4 days/week, and ≥5 days/week, respectively (p for trend < 0.01).

Table 2 presents the association between makeup use frequency and depressive symptoms. In Model 1, adjusted for age, medical history (diabetes mellitus, hypertension, and hyperlipidemia), education, paid work, living alone, BMI, MMSE score, and gait speed, participants who used makeup ≥5 days/week had lower odds of depressive symptoms than those who never used makeup (OR = 0.49; 95% CI = 0.33–0.73; *p* < 0.01). In Model 2, low social activity was independently associated with depressive symptoms (OR = 1.74; 95% CI = 1.22–2.49; *p* < 0.01), and frequent makeup use (≥5 days/week) remained associated with lower odds of depressive symptoms compared with never use (OR = 0.51; 95% CI = 0.34–0.77; *p* < 0.01).

**Table 2:** Association between frequency of makeup use and depressive symptoms adjusted for covariates.

Model 1				Model 2		
	OR	95% CI	p-value	OR	95% CI	p-value
Frequency of makeup use						
Never		Reference			Reference	
1–2/week	0.67	0.40, 1.11	0.126	0.68	0.40, 1.13	0.143
3–4/week	0.64	0.37, 1.07	0.097	0.64	0.37, 1.08	0.105
≥5/week	0.49	0.33, 0.73	<0.001	0.51	0.34, 0.77	0.001
Social activity						
High activity					Reference	
Low activity				1.74	1.22, 2.49	0.002

Abbreviations: CI = confidence interval, OR = odds ratio.

Model 1Adjusted for age, education, body mass index, hypertension, diabetes, hyperlipidemia, Mini-Mental State Examination, living alone, gait speed and paid work.

Model 2 Adjusted for age, education, body mass index, hypertension, diabetes, hyperlipidemia, Mini-Mental State Examination, living alone, gait speed, paid work and social activity.

Stratified analyses by social activity are shown in Table 3. Among participants with high social activity, makeup use ≥5 days/week was associated with lower odds of depressive symptoms compared with the never group among those with high social activity (OR = 0.49; 95% CI = 0.25–0.95). A similar association was observed among participants with low social activity, makeup use ≥5 days/week was also associated with reduced odds of depressive symptoms (OR = 0.54; 95% CI = 0.32–0.90).

**Table 3:** Odds ratios for depressive symptoms by frequency of makeup use in high and low social activity groups.

	High social activity			Low social activity		
	OR	95% CI	p-value	OR	95% CI	p-value
Never		Reference			Reference	
1–2/week	0.56	0.21, 1.38	0.227	0.73	0.39, 1.35	0.329
3–4/week	0.65	0.25, 1.54	0.345	0.62	0.31, 1.19	0.163
≥5/week	0.49	0.25, 0.95	0.034	0.54	0.32, 0.90	0.018

Abbreviations: CI = Confidence Interval, OR = Odds Ratio.

Adjusted for age, education, body mass index, hypertension, diabetes, hyperlipidemia, Mini-Mental State Examination, living alone, gait speed and paid work.

## 4 Discussion

To our knowledge, this is the first study to examine the association between makeup use frequency and depressive symptoms among community-dwelling older Japanese women. The findings indicate that participants who used makeup more frequently had a lower prevalence of depressive symptoms. In addition, higher makeup use frequency was associated with lower odds of depressive symptoms in both high and low social activity groups.

This study demonstrated that frequent makeup use was associated with a lower prevalence of depressive symptoms among community-dwelling older Japanese women. A previous cross-sectional study reported that makeup use was associated with a lower prevalence of mild depression among Brazilian women aged >30 years [7]. Similarly, a randomized controlled trial showed that increased makeup use resulted in sustained reductions in depressive symptoms, decreased cortisol levels, and improved self-image among Brazilian women [8]. Although these studies involved populations that differed in age and cultural background from those in the present study, their findings are consistent with the observed associations. One potential explanation for this association involves social interaction and participation. Mafra et al. [23] reported that women tend to use less makeup in non-social contexts, such as staying at home or exercising, and more makeup in social situations, including meeting friends or new people. Another study demonstrated that a beauty care program incorporating relaxation, makeup, and skincare helped maintain the frequency of going outdoors among older adults [9]. Large longitudinal studies have also shown that social interaction is associated with depressive symptoms [24], and multiple studies have reported that social participation is linked to a reduced risk of depressive symptoms among older adults [12,13]. Given that makeup use is often related to social engagement, social participation may partially explain the observed association between makeup use and depressive symptoms. In clinical or community screening, makeup use and other grooming behaviors may serve as non-intrusive signals. These findings may encourage clinicians to explore mood, social engagement, and self-care in more detail, particularly when habits appear to differ from an individual’s usual patterns. Furthermore, incorporating makeup use and grooming improvements as supplementary measures in mental health promotion programs in community settings may lead to engagement in these programs. However, because this was a cross-sectional study, causality could not be determined. Future longitudinal and intervention studies are necessary to determine whether assessing information regarding makeup use and personal grooming is useful for identifying depressive symptoms, and whether interventions such as applying makeup influence the reduction of depressive symptoms.

Cosmetics are defined as articles applied to the human body for cleansing, beautifying, promoting attractiveness, or altering physical appearance [6]. Makeup use may also be viewed as part of a broader behavioral process such as self-care capacity, adherence to cultural norms, and opportunities for social participation. Thus, makeup use can function as a behavioral marker of depressive symptoms. An interventional study conducted on Brazilian women reported a sustained reduction in depressive symptoms following increased makeup use [8]. This cross-sectional study did not establish any causality. Therefore, our findings cannot determine whether cosmetic behavior is a marker of depression or an indicator for intervention. Future longitudinal and interventional studies are needed to clarify directionality and causal mechanisms.

Stratified analyses showed that frequent makeup use was associated with a lower prevalence of depressive symptoms even among older adults with low social activity. This finding suggests that factors beyond social participation may contribute to the association. Neuroimaging studies using near-infrared spectroscopy (NIRS) have reported reduced prefrontal cortical hemodynamic responses in individuals with depression [25]. In a previous study using NIRS, 22 healthy women showed increased prefrontal oxyhemoglobin levels when viewing their own face with makeup compared with a bare-faced image [26]. Other research has shown that viewing one’s own face with mild beauty retouching activates the nucleus accumbens, a key region of the reward system [27]. These findings suggest that appearance-related behaviors, including makeup use, may influence brain activity related to reward and emotional processing. Nevertheless, the present study cannot determine causal mechanisms, and further studies are needed to clarify whether makeup-related behaviors directly influence depressive symptoms and to elucidate the underlying biological pathways.

This study has certain limitations. First, although participants were randomly selected and invited via direct mail, only those able to attend health check-ups were included, potentially resulting in a healthier study sample and underestimation of depressive symptoms. Second, the cross-sectional design precludes causal inference, and reverse causation is possible if depressive symptoms reduce motivation to use makeup. Third, this cross-sectional study used self-reported frequency of makeup use and depressive symptoms, and recall bias cannot be excluded despite the exclusion of participants with dementia or severe cognitive impairment. Fourth, the present study assessed only the frequency of makeup use and not the extent, types, or contexts of use (home vs. going out). Furthermore, our study evaluated the frequency of makeup use at a single time point and was unable to assess changes over time. Fifth, the present study recruited community-dwelling Japanese women aged 70 years and older, in accordance with our previous research [28]. Cultural differences and age groups may influence both makeup use habits and depressive symptoms. Therefore, caution is needed when applying our findings to other countries and age groups. Furthermore, to our knowledge, comparable population-based data on makeup use frequency among community-dwelling Japanese women aged ≥70 are limited and careful interpretation is necessary when considering its representativeness. Sixth, although an association was observed, the physiological mechanisms linking makeup use and depressive symptoms remain unclear. Future research will be necessary to clarify the physiological mechanisms that explain this association. Seventh, the study assessed makeup use as a behavioral practice and was not designed to evaluate any specific cosmetic products or brands. However, this study was conducted among community-dwelling older Japanese women, and the frequency of makeup use may differ across gender, age group, and culture. Therefore, caution is necessary when generalizing our findings to similar populations. Eighth, this was an observational study and no prior calculation of the sample size was performed. In the present study, we conducted a sub-analysis and reduced the sample size within each group, which may have affected the statistical power and precision. The results of this study should be interpreted with caution and should be considered exploratory. To clarify causality, future studies should include intervention studies with a priori sample size calculations. Finally, the present study failed to address other covariates (e.g., personality traits, duration of physical illness, genetic factors, lifetime socioeconomic status, and prior mental health history) related to makeup use and depressive symptoms.

In conclusion, frequent makeup use was associated with a lower prevalence of depressive symptoms among community-dwelling older Japanese women. Future research should evaluate whether interventions promoting cosmetic behaviors can prevent or reduce depressive symptoms and determine the populations and conditions under which such interventions are most effective.

## Acknowledgements

We thank the Nagoya City Offices for their assistance with participant recruitment. We also thank all participants who participated in the study.

## Informed consent

All participants provided informed consent before participation. The study protocol was approved by the Ethics Committee of the National Center for Geriatrics and Gerontology.

## Funding

This study was supported by the Japan Health Research Promotion Bureau (JH) Research Fund (JH-2019-(1)-1, 2024-B-05); the Six National Center Cohort Collaborative for Advancing Population Health Grant; the Research Funding for Longevity Sciences (21–16) from the National Center for Geriatrics and Gerontology; the National Institutes of Health Research Project Grant (R01 AG057548–01A1); and JSPS KAKENHI Grants JP22H03462 and JP23K24720. Additional financial support was provided through joint research with POLA Chemical Industries, Inc.

## Declaration of the use of generative AI and AI-assisted technologies in scientific writing and in figures, images and artwork

During the preparation of this manuscript, the authors used ChatGPT (OpenAI) to improve the readability and language of the manuscript, assist in sentence restructuring, and refine overall clarity. After using this tool/service, manuscript was subsequently reviewed by a professional English-language editing service. the authors reviewed and edited the content as needed and takes full responsibility for the content of the published article.

## Data statement

The datasets used and/or analyzed during the present study are available from the corresponding author on reasonable request.

## CRediT authorship contribution statement

**Yuto Kiuchi:** Writing – original draft, Methodology, Investigation, Formal analysis, Data curation, Conceptualization. **Sho Nakakubo:** Writing – review & editing, Formal analysis. **Shinnosuke Nosaka:** Writing – review & editing, Methodology, Investigation, Formal analysis, Data curation. **Yuka Misu:** Writing – review & editing, Methodology, Data curation, Conceptualization. **Kazuhei Nishimoto:** Writing – review & editing, Methodology, Investigation, Data curation. **Takamasa Gomi:** Writing – review & editing, Project administration, Methodology. **Shu Nishikori:** Writing – review & editing, Methodology. **Kanon Abe:** Writing – review & editing. **Tai Takemoto:** Writing – review & editing. **Hiroyuki Shimada:** Writing – review & editing, Project administration, Methodology, Investigation, Funding acquisition, Data curation, Conceptualization.

## Declaration of competing interest

The authors declare the following financial interests/personal relationships which may be considered as potential competing interests: This study was financially supported through a joint research agreement with POLA Chemical Industries, Inc. Yuto Kiuchi, Takamasa Gomi, Shu Nishikori, and Tai Takemoto are employees of POLA Chemical Industries, Inc. All other authors declare no conflicts of interest.

## References

[1] Byers A.L., Yaffe K., Covinsky K.E., Friedman M.B., Bruce M.L (2010). High occurrence of mood and anxiety disorders among older adults: the National Comorbidity Survey Replication. Arch Gen Psychiatry.

[2] Cai H., Jin Y., Liu R., Zhang Q., Su Z., Ungvari G.S. (2023). Global prevalence of depression in older adults: a systematic review and meta-analysis of epidemiological surveys. Asian J Psychiatr.

[3] Elser H., Horvath-Puho E., Gradus J.L., Smith M.L., Lash T.L., Glymour M.M. (2023). Association of early-, middle-, and late-life depression with incident dementia in a Danish cohort. JAMA Neurol.

[4] Penninx B.W., Guralnik J.M., Ferrucci L., Simonsick E.M., Deeg D.J., Wallace R.B (1998). Depressive symptoms and physical decline in community-dwelling older persons. JAMA.

[5] Schulz R., Beach S.R., Ives D.G., Martire L.M., Ariyo A.A., Kop W.J (2000). Association between depression and mortality in older adults: the cardiovascular health study. Arch Intern Med.

[6] US Food and Drug Administration. How U.S. Law defines cosmetic and cosmetic product, https://www.fda.gov/cosmetics/cosmetics-laws-regulations/cosmetics-us-law [accessed 12/17 2025].

[7] Vecoso M.C., Bagatin E., Fonseca F.L.A., Andreo-Filho N., Lopes P.S., Leite-Silva V.R (2023). Association between frequent use of makeup and presence of depressive symptoms-population-based observational study, including 2400 participants. Dermatol Ther.

[8] Vecoso M.C., Zalla S., Andreo-Filho N., Lopes P.S., Bagatin E., Fonseca F.L.A. (2024). Effect of makeup use on depressive symptoms: an open, randomized and controlled trial. Dermatol Ther.

[9] Kawai H., Inomata T., Otsuka R., Sugiyama Y., Hirano H., Obuchi S. (2016). [The effectiveness of beauty care on self-rated health among community-dwelling older people]. Nihon Ronen Igakkai Zasshi.

[10] Tadokoro K., Yamashita T., Sato J., Omote Y., Takemoto M., Morihara R. (2022). Chronic beneficial effect of makeup therapy on cognitive function of dementia and facial appearance analyzed by artificial intelligence software. J Alzheimers Dis.

[11] Umberson D., Montez J.K (2010). Social relationships and health: a flashpoint for health policy. J Health Soc Behav.

[12] Shiba K., Torres J.M., Daoud A., Inoue K., Kanamori S., Tsuji T. (2021). Estimating the impact of sustained social participation on depressive symptoms in older adults. Epidemiology.

[13] Takemura Y., Inoue K., Sato K., Haseda M., Shiba K., Kondo N. (2025). Social participation and depressive symptoms among older adults. JAMA Netw Open.

[14] Shimada H., Doi T., Lee S., Makizako H., Chen L.K., Arai H. (2018). Cognitive frailty predicts incident dementia among community-dwelling older people. J Clin Med.

[15] Folstein M.F., Folstein S.E., McHugh P.R (1975). “Mini-mental state”. J Psychiatr Res.

[16] Yesavage J.A (1988). Geriatric depression scale. Psychopharmacol Bull.

[17] Misu Y., Tsutsumimoto K., Kiuchi Y., Nishimoto K., Ohata T., Shimada H. (2025). Coexistence of somatic and psychological symptoms of depression among community-dwelling older adults is associated with the incidence of dementia. J Alzheimers Dis.

[18] Lyness J.M., Noel T.K., Cox C., King D.A., Conwell Y., Caine E.D (1997). Screening for depression in elderly primary care patients. A comparison of the center for epidemiologic studies-depression scale and the geriatric depression scale. Arch Intern Med.

[19] van Marwijk H.W., Wallace P., de Bock G.H., Hermans J., Kaptein A.A., Mulder J.D (1995). Evaluation of the feasibility, reliability and diagnostic value of shortened versions of the geriatric depression scale. Br J Gen Pract.

[20] Doi T., Tsutsumimoto K., Nakakubo S., Kurita S., Ishii H., Shimada H. (2022). Associations between Active mobility Index and disability. J Am Med Dir Assoc.

[21] Makizako H., Shimada H., Doi T., Tsutsumimoto K., Suzuki T. (2015). Impact of physical frailty on disability in community-dwelling older adults: a prospective cohort study. BMJ Open.

[22] Shimoda T., Tomida K., Nakajima C., Kawakami A., Doi T., Shimada H. (2024). Impact of working time and industry type on disability incidence among older Japanese adults. Discover Public Health.

[23] Mafra A.L., de Moraes Y.L., Varella M.A.C., Valentova J.V (2025). Makeup usage in women is positively associated to narcissism and extraversion but negatively to psychopathy. Arch Sex Behav.

[24] Misawa J., Kondo K. (2019). Social factors relating to depression among older people in Japan: analysis of longitudinal panel data from the AGES project. Aging Ment Health.

[25] Takizawa R., Fukuda M., Kawasaki S., Kasai K., Mimura M., Pu S., al et (2014). Neuroimaging-aided differential diagnosis of the depressive state. Neuroimage.

[26] Ikeuchi M., Saruwatari K., Takada Y., Shimoda M., Nakashima A., Inoue M. (2014). Evaluating “cosmetic therapy” by using near-infrared spectroscopy. World J Neurosci.

[27] Ota C., Nakano T. (2021). Neural correlates of beauty retouching to enhance attractiveness of self-depictions in women. Soc Neurosci.

[28] Tsutsumimoto K., Doi T., Makizako H., Hotta R., Nakakubo S., Makino K. (2017). The association between anorexia of aging and physical frailty: results from the national center for geriatrics and gerontology's study of geriatric syndromes. Maturitas.

